# Synthesis and Characterization of Novel Triphenylamine—Containing Electrochromic Polyimides with Benzimidazole Substituents

**DOI:** 10.3390/molecules28052029

**Published:** 2023-02-21

**Authors:** Kuangguo Yan, Haiquan Chen, Chenjie Zhu, Zhao Ke, Dongwu Li, Mengxia Wang, Fengna Dai, Youhai Yu

**Affiliations:** Center for Advanced Low-Dimension Materials, State Key Laboratory for Modification of Chemical Fibers and Polymer Materials, College of Material Science and Engineering, Donghua University, Shanghai 201620, China

**Keywords:** benzimidazole, two-step polymerization, polyimide films, electrochromic materials

## Abstract

Two novel electrochromic aromatic polyimides (named as TPA-BIA-PI and TPA-BIB-PI, respectively) with pendent benzimidazole group were synthesized from 1,2-Diphenyl-N,N′-di-4-aminophenyl-5-amino-benzimidazole and 4-Amino-4′-aminophenyl-4″-1-phenyl-benzimidazolyl-phenyl-aniline with 4,4′-(hexafluoroisopropane) phthalic anhydride (6FDA) via two-step polymerization process, respectively. Then, polyimide films were prepared on ITO-conductive glass by electrostatic spraying, and their electrochromic properties were studied. The results showed that due to the π-π* transitions, the maximum UV–Vis absorption bands of TPA-BIA-PI and TPA-BIB-PI films were located at about 314 nm and 346 nm, respectively. A pair of reversible redox peaks of TPA-BIA-PI and TPA-BIB-PI films that were associated with noticeable color changed from original yellow to dark blue and green were observed in the cyclic voltammetry (CV) test. With increasing voltage, new absorption peaks of TPA-BIA-PI and TPA-BIB-PI films emerged at 755 nm and 762 nm, respectively. The switching/bleaching times of TPA-BIA-PI and TPA-BIB-PI films were 13 s/16 s and 13.9 s/9.5 s, respectively, showing that these polyimides can be used as novel electrochromic materials.

## 1. Introduction

Electrochromism, a phenomenon of material changing its color or opacity under applied voltage, was first discovered by Platt [1]. Since Deb S.K reported the first electrochromic material, WO_3_ [2], electrochromic materials have received great attention due to high optical contrast, optical tunability and reversibility and have been widely used in the fields of smart window, ink printing, electronic paper and so on [3,4,5,6,7]. For decades, various electrochromic materials, including inorganic metal oxides, small organic materials and conductive polymers have been developed [8,9,10,11,12].

As electrochromic materials, donor-acceptor (D-A) type conjugated polymers endow the polymers with good electroactivity by alternately linking electron-rich and electron-deficient groups on the molecular backbone to induce electron transfer [13]. By selecting different donors and acceptors and adjusting the conjugation length, conjugated polymers with adjustable colors can be designed [14,15].

A high-performance polyimide (PI) [16,17] is a kind of conjugated polymer which has been widely used in electronic fields, aerospace and military aspects due to their excellent thermal properties, mechanical properties and chemical corrosion resistance. However, the rigid skeleton structure and strong intermolecular force greatly reduce the solubility of PIs, increasing their process difficulty [18,19]. To solve this problem, flexible groups, twisted asymmetric structures and large volume side groups have been introduced on the polymer skeleton to improve the solubility of PI [20,21,22,23,24,25].

Triphenylamine (TPA) with a propeller-like structure can disrupt the coplanarity in main chains of polymer and increase the free volume of the polymer chain to improve the solubility of PIs in common solvents. Meanwhile, when an external voltage is applied, TPA and its derivatives with nitrogen atoms as the center can be easily oxidized to cation radicals (TPA^+^), accompanied with visible color change [26,27,28]. Therefore, they are often used as donor units to combine with electron-withdrawing groups to form D-A type conjugated polymers to induce intramolecular charge transfer [29,30,31,32]. Based on this, molecular design methods are used to introduce different donor units into the triphenylamine and combine with different acceptor units, thereby adjusting the band gap to regulate the color change in electrochromic materials [33,34,35]. In addition, electrochromic materials with electron-donating groups in the para position of triphenylamine often show lower redox potential and better electrochemical stability [36,37]. Benzimidazole is a nitrogen-containing aromatic five-membered heterocyclic structural molecule. Due to their good electron transfer ability, benzimidazole and its derivatives are widely used in organic light-emitting diodes [38,39]. Cai et al. introduced benzimidazole as a donor unit into diamine monomer and finally synthesized polyimides with electrochromic properties. The results showed that the polyimide with benzimidazole unit had lower redox potential and excellent electrochemical stability compared with other donor units [40].

In this work, benzimidazole derivatives with different structures were introduced into the diamine monomer as the donor unit and combined with the imide ring as the acceptor unit to form a polyimide with D-A conjugated structure, which induced charge transfer between the diamine monomer and the dianhydride monomer, while the polyimides with two structures had two color changes due to different band gaps. In addition, we tried to introduce benzimidazole derivatives to the para position of triphenylamine to reduce the redox potential of polyimide and improve electrochemical stability. 2,4-dinitrodiphenylamine and 4-nitro-o-phenylenediamine were used as raw materials to prepare 2-diphenyl-N,N′-di-4-nitrophenyl-5-amine benzimidazole (TPA-BIA-NO_2_) and 4-Nitro-4′-nitrophenyl-4″-1-phenyl-benzimidazolyl-phenyl-aniline (TPA-BIB-NO_2_) through chloride reaction, acetic acid ring closure and nucleophilic substitution, respectively. Afterward, two diamine monomers 1,2-diphenyl-N,N′-di-4-aminophenyl-5-amine-benzimidazole and 4-amino-4′-aminophenyl-4″-1-Phenyl-benzimidazolyl-phenyl-aniline were synthesized by the palladium carbon-hydrazine hydrate catalytic reduction, and we named these two monomers as TPA-BIA-NH_2_ and TPA-BIB-NH_2_, respectively. These two diamine monomers were polymerized with 6FDA into electrochromic polyimides, which were named as TPA-BIA-PI and TPA-BIB-PI, respectively. The results showed that both D-A type structural polyimides had good solubility and thermal stability as well as electrochromic properties.

## 2. Results and Discussion

### 2.1. Monomer Synthesis

**Scheme 1:**
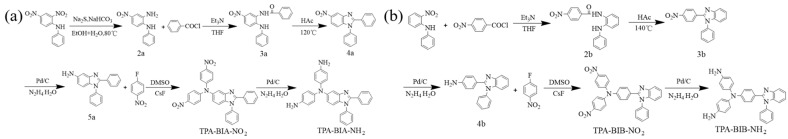
Synthetic route of TPA-BIA-NH_2_ (**a**) and TPA-BIB-NH_2_ (**b**).

**Figure 1:**
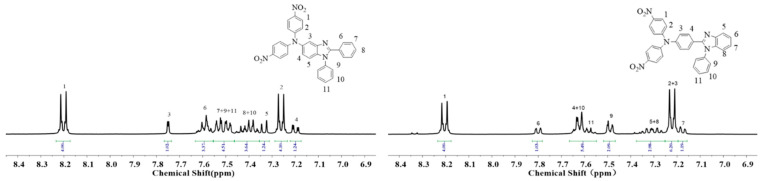
^1^H-NMR spectra of TPA-BIA-NO_2_ and TPA-BIB-NO_2_.

As shown in Scheme 1, two new diamines containing benzimidazole groups were synthesized step by step through a multi-step method. First, we synthesized TPA-BIA-NO_2_ and TPA-BIB-NO_2_ by nucleophilic substitution. The ^1^H NMR spectra of TPA-BIA-NO_2_ and TPA-BIB-NO_2_ were illustrated in Figure 1. 

**Figure 2:**
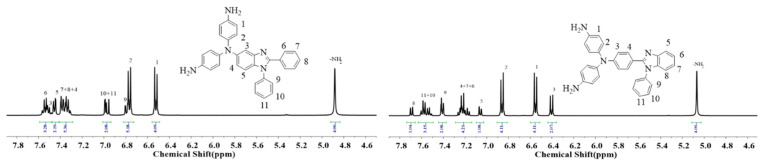
^1^H-NMR spectra of TPA-BIA-NH_2_ and TPA-BIB-NH_2_.

Then, it was reduced to TPA-BIA-NH_2_ and TPA-BIB-NH_2_ by Pd/C and hydrazine hydrate. The ^1^H NMR spectra of TPA-BIA-NH_2_ and TPA-BIB-NH_2_ were illustrated in Figure 2. It can be seen that the amine peaks of TPA-BIA-NH_2_ and TPA-BIB-NH_2_ appeared at about 4.9 ppm and 5.1 ppm. Additionally, the two-dimensional hydrogen spectrum showed that the chemical shift distribution of the H atom at each position, and the attribution were clear (Figures S1 and S2). In addition, the molecular structures were further confirmed and characterized by infrared spectroscopy. As seen in Figures S3 and S4, the C-N vibration peak of TPA-BIA-NH_2_ appeared at 1381 cm^−1^; the characteristic absorption peak of the imidazole ring appeared at 1323 cm^−1^, while the characteristic absorption bands at 3318 and 3328 cm^−1^ were attributed to amino group. At the same time, the C-N vibration peak of TPA-BIB-NH_2_ appeared at 1374 cm^−1^; the characteristic absorption peak of the imidazole ring appeared at 1332 cm^−1^, while the characteristic absorption bands at 3364 and 3448 cm^−1^ were attributed to amino group, which proved that we successfully synthesized two diamine monomers.

### 2.2. Polymer Synthesis

**Figure 3:**
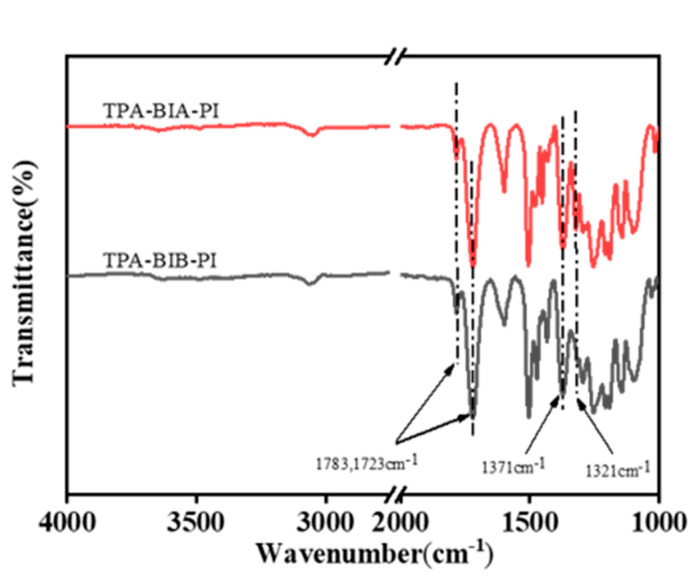
FT-IR spectra of the polyimides.

TPA-BIA-NH_2_ and TPA-BIB-NH_2_ were used as two diamine monomers to synthesize a new polyimide with pendant benzimidazole groups through a series of polycondensation reactions with commercial 6FDA. These polymerization processes were uniform, and all formed high-viscosity polymer solutions. As shown in Figure 3, C=O asymmetric stretching vibration peaks and symmetric stretching vibration peaks appeared at 1783 cm^−1^ and 1723 cm^−1^; C-N stretching vibration peak appeared at 1371cm^−1^; the characteristic absorption peak of the imidazole ring appeared at 1321cm^−1^. In the range of 3200–3450 cm^−1^, there was no absorption peak of polyamide acid, which proved that all polyimides have been successfully synthesized and completely imidized [41].

### 2.3. Basic Properties

To meet the requirement of practical application, EC polymers should have good processability and solubility. The solubility of the two polyimides was investigated by dissolving 10 mg of the sample in 1 mL of organic solvent. All results were shown in Table 1. Due to the introduction of a huge twisting group, both TPA-BIA-PI and TPA-BIB-PI could be dissolved in polar solvents such as NMP, DMAc, DMF and DMSO. In low-polar solvents such as THF and CHCI_3_, TPA-BIA-PI and TPA-BIB-PI also had good solubility, mainly because the bulky side groups further increased the polymer chain. The free volume between them allowed the solvent molecules to further enter the polymer chain. Excellent solubility was beneficial to the later coating and better processability to prepare large-area devices. In addition, the number-average molecular weights (Mw) and polydispersity (PDI) were tested with GPC; these two polymers both have high number-average molecular weights (Mn). The PDI of TPA-BIA-PI and TPA-BIB-PI were found to be 1.2 and 2.6, respectively. The reason why the PDI of TPA-BIB-PI is much larger than that of TPA-BIA-PI can be attributed to the fact that the high apparent viscosity affects the uniformity of the mixture during the polymerization process, which will lead to differences in molecular weight distribution and thus deviation from the desired polycondensation [37].

### 2.4. Thermal Properties

**Figure 4:**
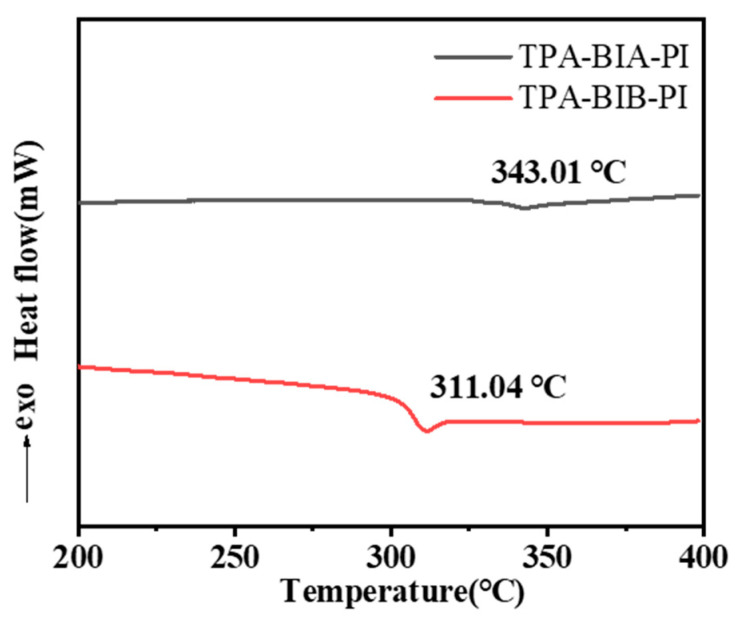
DSC curves of the polyimides.

**Figure 5:**
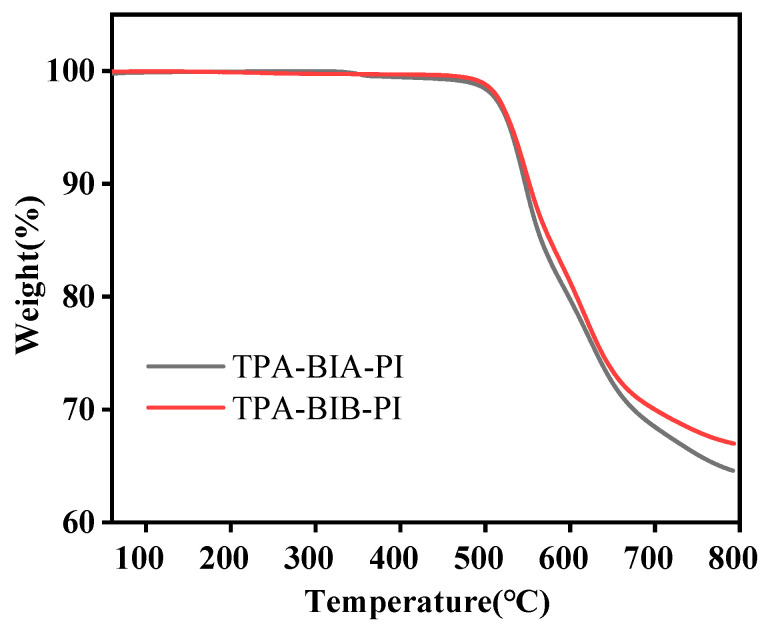
TGA curves of the polyimides.

In practical applications such as smart windows and car rearview mirrors, materials are required to have sufficient thermal stability to face uneven rising temperatures. We evaluated the thermal performance of TPA-BIA-PI and TPA-BIB-PI by TGA and DSC. The data were shown in Table 2. The glass transition temperature was related to the rigidity of the polymer. As shown in Figure 4, the T*_g_* values of the two polyimides were above 310 °C. In contrast, TPA-BIA-PI had higher value of T*_g_* due to larger spatial site resistance, so that limited the rotation of its molecular chain. As shown in Figure 5, the 5% thermal decomposition temperatures of the two polyimides are 529 °C and 531 °C, respectively. Under a nitrogen atmosphere at 800 °C, the carbon residual rates of the two polymers were about 65%. This is mainly due to the high aromatic content in their structure.

### 2.5. Optical Properties

**Figure 6:**
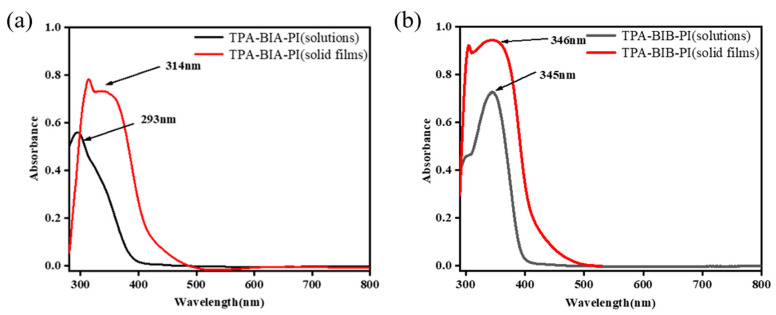
UV–vis absorption spectra of TPA-BIA-PI and TPA-BIB-PI in NMP solutions (**a**) and solid films (**b**).

**Figure 7:**
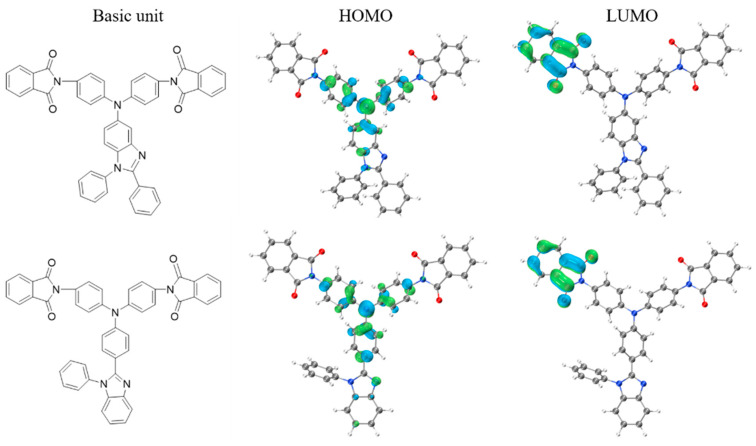
Calculated molecular orbitals of the model compounds (TD-DFT method at the B3LYP/6-31G (d, p)).

Ultraviolet–visible spectroscopy was used to study the optical properties of TPA-BIA-PI and TPA-BIB-PI, and the results were shown in Figure 6 and Table 3. As shown in Figure 6, in the NMP solution, the maximum absorption peak of TPA-BIA-PI appeared at 293 nm, and the maximum absorption peak of TPA-BIB-PI appeared at 345 nm. In the solid film, the maximum absorption peaks of TPA-BIA-PI and TPA-BIB-PI were 318 nm and 347 nm, respectively, which is mainly attributed to the transition of the “triphenylamine-benzimidazole” group π-π*. The cut-off wavelengths of TPA-BIA-PI and TPA-BIB-PI were 465 nm and 471 nm, respectively. From this, we could estimate that the optical energy band gap is approximately 2.67 eV and 2.63 eV. To better explain this phenomenon, we use TD-DFT calculations to clarify the electronic transition properties of the two “triphenylamine-benzimidazole” structures. As seen in Figure 7, we used the Gaussian 16 program to calculate the charge distribution in the TPA-BIA-PI and TPA-BIB-PI molecules at the TD-DFT/b3lyp/6-311++g(d, p) level [42] and used multiwfn 3.7 software [43] for further analysis. The calculated HOMO-LUMO energies differ slightly from those determined from the absorption spectra and cyclic voltammograms, but the observed trends were roughly consistent with the actually tested trends. In this paper, the electrochromic phenomenon is produced by the oxidation of nitrogen atoms in triphenylamine to cationic radicals. In the molecular backbone of TPA-BIA-PI and TPA-BIB-PI with D-A structure, the donor unit “triphenylamine-benzimidazole” and the acceptor unit “imide ring” are alternately connected and have charge transfer capability, thus giving polyimide good electrical activity. As can be seen from the electron cloud distribution in Figure 7, the highest occupied orbital (HOMO) electrons of the polyimides are concentrated in the “triphenylamine-benzimidazole” donor unit, while the lowest unoccupied molecular orbital (LUMO) electrons are concentrated in the “imide ring” acceptor unit. This means that the charge transfer between the electron donor unit and electron acceptor unit occurs during the transition from the ground state to the excited state of the polyimide. In addition, these two polyimides turn the films into colors of corresponding wavelengths due to their different energy band structures.

### 2.6. Electrochemical and Electrochromic Properties

**Figure 8:**
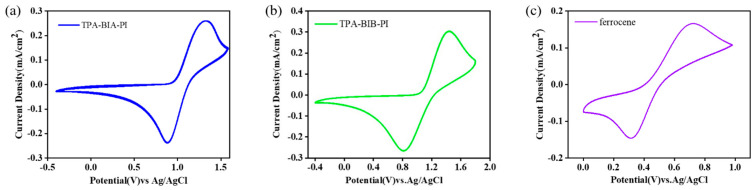
Cyclic voltametric diagrams of the polymers (**a**) TPA-BIA-PI, (**b**) TPA-BIB-PI, (**c**) ferrocene in CH_3_CN containing 0.1M TBAP at a scan rate of 100 mV·s^−1^.

**Figure 9:**
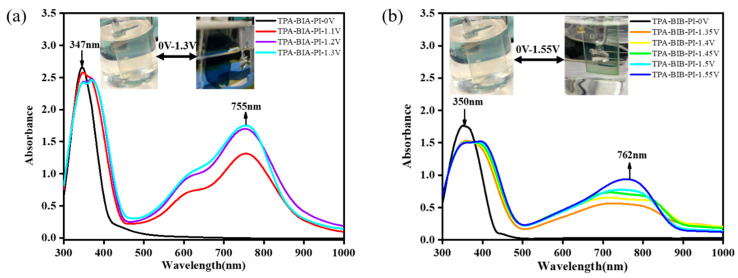
On the ITO–coated glass substrate (in CH_3_CN, 0.1M TBAP is used as the supporting ele–trolyte) the spectral changes in TPA-BIA-PI (**a**) and TPA-BIB-PI (**b**) with increasing voltage.

**Figure 10:**
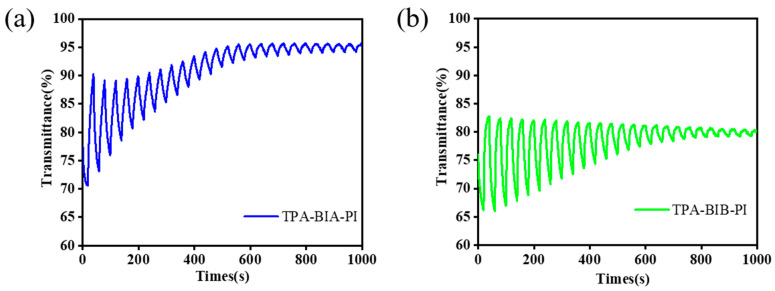
Optical switching between −0.4 and 1.6 V of the polyimide TPA-BIA-PI (**a**) film and −0.4 and 1.8 V of the polyimide TPA-BIB-PI (**b**) on the ITO-coated glass substrate in 0.1M TBAP/CH_3_CN with a cycle time of 20 s.

**Figure 11:**
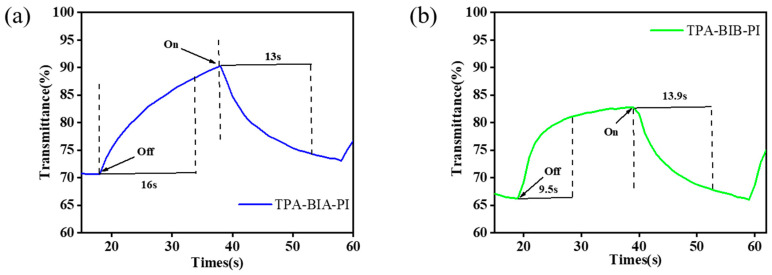
Optical switching time of TPA-BIA-PI at 755 nm (**a**) and TPA-BIB-PI at 762 nm (**b**).

The electrical properties of TPA-BIA-PI and TPA-BIB-PI were studied by three-electrode cyclic voltammetry. Polyimide films were sprayed on the ITO substrate as the working electrode. In Figure 8, both polyimides had a pair of reversible redox couples. The oxidation half-wave potentials of TPA-BIA-PI and TPA-BIB-PI were 1.2 eV and 1.5 eV, respectively. HOMO of the two polyimides was calculated through the ionization potential and E_1/2_ with ferrocene (4.8 eV); LUMO was calculated through the formula HOMO = LUMO − E*_g_*; these electrochemical data were summarized in Table 3. In the CV measurement, it can be observed that the two polyimide films had obvious color changes with the CV scan. To further study the electrochromic properties of TPA-BIA-PI and TPA-BIB-PI, spectroelectrochemical techniques were performed. It can be seen from Figure 9 that the characteristic absorption peak of TPA-BIA-PI at 347 nm gradually decreased during the 0–1.3V electrooxidation process, and a new peak appeared at 755 nm, which is mainly due to the central nitrogen atom becoming the cationic free radicals. At the same time, the color of TPA-BIA-PI had also changed from light yellow neutral state to dark blue oxidation state. The absorption peak at 350 nm of TPA-BIB-PI gradually decreased during the electrooxidation process at 0–1.55 V, and a new peak appeared at 762 nm; the color changed from yellow to green. The charge transfer mechanism of polyimide films producing electrochromic phenomenon was shown in Figure S5. Using electrochemical workstation and ultraviolet–visible light spectroscopy, through the square wave potential between the neutral state and the oxidation state, the change in transmittance with time at a fixed wavelength and the cycle stability of the film were observed at the same time. The typical switching behaviors of TPA-BIA-PI and TPA-BIB-PI were performed by applying square wave voltages. As shown in Figure 10, it can be seen from the figure that the transmission ratio of TPA-BIA-PI and TPA-BIB-PI had a significant decay with time. After 20 cycles, the optical contrasts of TPA-BIA-PI and TPA-BIB-PI were 20% and 44% of the originals, respectively. Switch response time is also an important parameter of electrochromism, which is defined as the voltage application time of 90% of the maximum spectral change value. It can be seen that the coloring time of TPA-BIA-PI at 755 nm was 13 s, and the blenching time was 16 s while coloring time of TPA-BIB-PI at 762 nm was 13.9 s, and the blenching time was 9.5 s (Figure 11). As seen in Table 3, TPA-BIA-PI and TPA-BIB-PI have higher oxidation potential and half-wave potential, which may be related to the electron-giving ability of two benzimidazole derivatives, and the higher oxidation potential and initial potential will affect the cyclic stability of the material in the electrochromic process. In addition, due to the strong electron-absorbing ability of carbonyl in the imide ring, it is easy to produce peroxidation, which will cause irreversible electrical deactivation of the material, thus reducing the electrochemical stability [44].

Based on this, we consider replacing the imide bond with amide bond or sulfone group to reduce the redox potential of the material and improve the cyclic stability of electrochromism in the future.

## 3. Experimental

### 3.1. Materials

2,4-Dinitrodiphenylamine and 4-nitro-o-phenylenediamine are used as starting monomers for two synthetic routes. Among them, 2,4-dinitrodiphenylamine (TCI), 4-Nitrobenzoyl chloride (TCI), 4-nitro-1,2-phenylenediamine (Shanghai Energy Chemical Co., Ltd., Shanghai, China), benzoyl chloride (Shanghai Aladdin Chemical Co., Ltd., Shanghai, China) and pyridine (Shanghai Aladdin Chemical Co., Ltd.) are all used as accepted. Pd/C and acetic anhydride were procured from Sinopharm Chemical Reagent Beijing Co., Ltd. Cesium fluoride, triethylamine, 4-Fluoronitrobenzene, ethanol, tetrahydrofuran, acetic acid, dimethyl sulfoxide (DMSO) and N,N-dimethylacetamide (DMAc) were procured from Shanghai Titan Scientific Co., Ltd. In addition, ethanol, tetrahydrofuran, acetic acid, dimethyl sulfoxide (DMSO) and N,N-dimethylacetamide (DMAc) were purified by vacuum distillation and stored over 4 Å molecular sieves before use. The synthesized diamine monomer and commercial 4,4′-(Hexafluoroisopropylidene) phthalic anhydride were treated in a vacuum oven at 180 °C for water removal before use.

### 3.2. Synthesis of Compounds

#### 3.2.1. Synthesis of 1,2-Diphenyl-N,N′-di-4-nitrophenyl-5-amine benzimidazole (TPA-BIA-NO_2_)

A mixture of 1,2-Diphenyl-5-aminobenzimidazole (8.8 g, 31 mmol), 4-Fluoronitrobenzene (13.1 g, 93 mmol) and cesium fluoride (16.5 g, 108 mmol) in 90 mL of DMSO was heated and stirred at 120 °C for 14 h. After cooling to room temperature, the mixed solution was poured into 90 mL of water and continuously stirred until the yellow crude product was completely precipitated. Then, the yellow crude product was recrystallized with dioxane/water to obtain the desired dinitro compound. FT-IR (ATR): 1576, 1333cm^−1^ (−NO_2_ stretch). ^1^H NMR (400 MHz, DMSO-*d*_6_, δ, ppm): 8.25–8.16 (m, 2H), 7.65–7.45 (m, 3H), 7.48–7.16 (m, 4H), 3.57 (s, 2H), 3.38 (s, 9H), 1.29 (s, 1H), 1.24 (s, 1H).

#### 3.2.2. Synthesis of 1,2-Diphenyl-N,N′-di-4-aminophenyl-5-amino-benzimidazole (TPA-BIA-NH_2_)

1,2-Diphenyl-N,N′-di-4-nitrophenyl-5-amine benzimidazole (5 g, 10 mmol), 0.5 g Pd/C mixture were dissolved into 50 mL DMSO under stirring, and the temperature was raised to 80 °C, and then hydrazine hydrate (5.9 g, 119 mmol) was added dropwise to the mixed solution and stirred for 4 h. Then, the Pd/C was filtered, and the mixed solution was slowly poured into 150 mL of water and filtered. Finally, the tea green diamine monomer was obtained. FT-IR (ATR): 3331 cm^−1^, 3219cm^−1^ (−NH_2_ stretch). ^1^H NMR (400 MHz, DMSO-*d*_6_, δ, ppm): 7.59–7.46 (m, 3H), 7.50–7.42 (m, 2H), 7.42–7.28 (m, 5H), 7.02–6.93 (m, 2H), 6.83–6.72 (m, 5H), 6.57–6.48 (m, 4H), 4.88 (s, 4H), 3.57 (s, 2H), 1.24 (s, 1H).

#### 3.2.3. Synthesis of 4-Nitro-4′-nitrophenyl-4″-1-phenyl-benzimidazolyl-phenyl-aniline (TPA-BIB-NO_2_)

A mixture of-phenyl-2-4-aminophenyl-benzimidazole (13.7 g, 48 mmol), 4-Fluoronitrobenzene (20.3 g, 144 mmol) and cesium fluoride (25.5 g, 168 mmol) in 140mL of DMSO was heated and stirred at 120 °C for 14 h. After cooling to room temperature, the mixed solution was put into 180 mL of water and continuously stirred until the yellow crude product was completely precipitated. Then, the yellow crude product was recrystallized with DMSO to obtain the ideal dinitro compound. FT-IR (ATR): 1500, 1334 cm^−1^ −NO_2_ stretch). ^1^H NMR (400 MHz, DMSO-*d*_6_, δ, ppm): 8.25–8.16 (m, 4H), 7.84–7.76 (m, 1H), 7.68–7.53 (m, 5H), 7.53–7.45 (m, 2H), 7.41–7.24 (m, 2H), 7.28–7.14 (m, 8H), 3.39 (s, 10H), 2.55 (s, 1H), 1.24 (s, 1H).

#### 3.2.4. Synthesis of 4-Amino-4′-aminophenyl-4″-1-phenyl-benzimidazolyl-phenyl-aniline (TPA-BIB-NH_2_)

4-Nitro-4′-nitrophenyl-4″-1-phenyl-benzimidazolyl-phenyl-aniline (8 g, 15 mmol), 0.8 g Pd/C mixture were dissolved into 80 mL of DMSO and stirred. The temperature was raised to 80 °C, and then hydrazine hydrate (9.5 g, 190 mmol) was added dropwise to the mixed solution and stirred for 4 h. The mixed solution was slowly poured into 160 mL of water and suction filtered and finally obtained the yellow diamine monomer. FT-IR (ATR): 3448, 3364cm^−1^ (−NH_2_ stretch). ^1^H NMR (400 MHz, DMSO-*d*_6_, δ, ppm): 7.69 (dt, *J* = 8.0, 0.9 Hz, 1H), 7.64–7.54 (m, 2H), 7.57–7.48 (m, 1H), 7.45–7.37 (m, 2H), 7.29–7.14 (m, 4H), 7.06 (dt, *J* = 7.8, 1.1 Hz, 1H), 6.90–6.82 (m, 4H), 6.60–6.51 (m, 4H), 6.45–6.36 (m, 2H), 5.07 (s, 4H).

### 3.3. Synthesis of Polyimides TPA-BIA-PI and TPA-BIB-PI

**Scheme 2:**
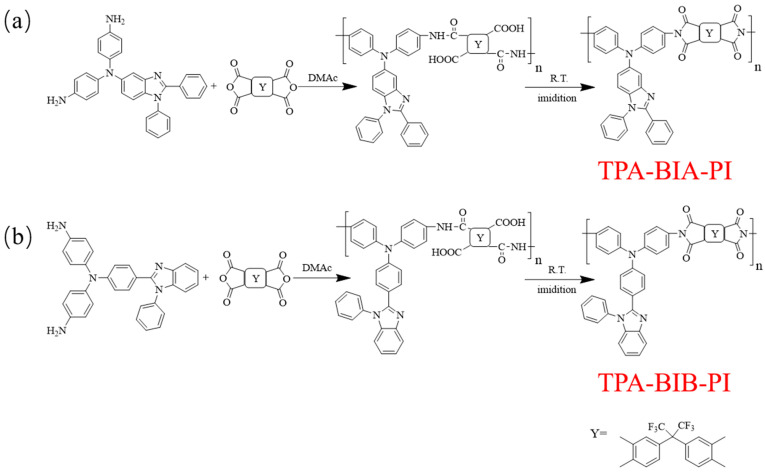
Synthesis of TPA-BIA-PI (**a**) and TPA-BIB-PI (**b**).

Two kinds of diamine monomers TPA-BIA-NH_2_ and TPA-BIB-NH_2_ and 6FDA were successfully polymerized into two polyimides TPA-BIA-PI and TPA-BIB-PI through the chemical imine method. Scheme 2 gives a typical polymerization process. In a nitrogen atmosphere, a mixture of diamine monomer (0.2503 g, 0.5 mmol) and dianhydride monomer (0.2299 g, 0.5 mmol) was dissolved in 1.4406 g of DMAc and stirred at room temperature for 8 h to polymerize into a polyamide acid solution with a solid content of 25%. After that, 0.4702 g and 0.4227 g of pyridine and acetic anhydride were sequentially added to the polyamide acid solution and stirred at room temperature for 10 h to further ring-closure polymerization to form a polyimide solution. Finally, the viscous polymer solution was slowly poured into ethanol and stirred to obtain a bright yellow fibrous precipitate. Then, the obtained precipitate was thoroughly washed with a mixed solution of water and ethanol. Then, it was vacuum dried at 100 °C. The polymer was finally reprecipitated twice with DMAc/ethanol for further purification.

### 3.4. Preparation of Polyimide Films

The preparation of the polymer film was as follows. A 0.6 g sample was dissolved in 10 mL of DMAc to form a homogenous solution. Then, the polymer solution was evenly sprayed onto the ITO glass by electrostatic spraying. The ITO substrates were vacuumed overnight at 150 °C [45].

### 3.5. Measurements

Proton nuclear magnetic resonance spectra (^1^H NMR) were obtained on a Bruker 600 AVANCE III spectrometer, in which dimethyl sulfoxide-d6 was used as the solvent. The inherent viscosities (η_inhs_) were measured with an Ubbelohde viscometer at 25 ± 0.1 °C using DMAc as the solvent. Fourier transform infrared (FTIR) spectra were recorded by a Nicolet 6700 infrared spectrometer with the ATR accessory. The resolution is 4 cm^−1^, and the range is 4000–400cm^−1^. Differential scanning calorimetric (DSC) analysis was performed on a TA instrument DSC 250 at a scanning rate of 10 °C/min in a nitrogen flow of 50 mL/min. The thermogravimetric analysis (TGA) was assessed using Discovery TGA 550 with a constant heating rate of 10 °C/min^−1^ under nitrogen. Electrochemical measurements were characterized by a CHI660e electrochemical analyzer. Cyclic voltammetry (CV) was performed with a three-electrode system. The polymer solution was sprayed on the ITO glass substrate as the working electrode. The platinum sheet was used as the counter electrode, and the Ag/AgCl, KCI (sat.) was used as the reference electrode. Ultraviolet–visible (UV–vis) spectra were measured using a PerkinElmer Lambda 950 spectrophotometer [46].

## 4. Conclusions

Based on 1,2-Diphenyl-N,N′-di-4-aminophenyl-5-amino-benzimidazole and 4-Amino-4′-aminophenyl-4″-1-phenyl-benzimidazolyl-phenyl-aniline, two novel polyimides containing TPA and Benzimidazole derivatives (TPA-BIA-PI and TPA-BIB-PI) were successfully synthesized via chemical imidization reaction. The maximum UV–Vis absorption bands appeared in the range of 310–350 nm for the solid films. TPA-BIA-PI and TPA-BIB-PI films revealed electrochemical behavior with high optical transmittance contrast of coloration changed from neutral yellow state to dark blue and green fully oxidized state. New absorption peaks emerged at 755 nm and 762 nm with increasing voltage in the UV–Vis–NIR spectrum, respectively. In addition, excellent solubility and thermal stability suggest that two novel polyimides have great potential in practical applications.

## Figures and Tables

**Table 1:** Inherent Viscosities, Molecular Weights and Solubilities of the Polyimides.

			Solvent ^a^					GPC ^c^ (×10^4^ g/mol)		
Polymer	NMP	DMAc	DMF	DMSO	THF	CHCl_3_	*η*_inh_^b^	M_w_	M_n_	PDI
TPA-BIA-PI	++	++	++	++	++	++	0.7	15.3	12.7	1.2
TPA-BIB-PI	++	++	++	+−	++	++	0.5	15.0	5.8	2.6

^a^ Qualitative solubilities were tested with 10 mg of polymers in 1 mL of solvent. ++, soluble at room temperature; +, soluble on heating; +−, partially soluble; −, insoluble even on heating. ^b^ Inherent viscosities were measured at a concentration of 0.5 g/dL in DMAc at 25 °C. ^c^ Relative to polystyrene standard, using DMF as the eluent.

**Table 2:** Thermal properties of the Polyimides.

Polymer	T*_g_* ^a^	T*_d_*_5%_^b^	R*_w_*_800_(%) ^c^
TPA-BIA-PI	343	529	64
TPA-BIB-PI	311	531	67

^a^ Obtained at the baseline shift in the second heating DSC traces, with a heating rate of 10 °C/min under N_2_. ^b^ Decomposition temperature at which a 10% weight loss was recorded via TGA at a heating rate of 10 °C/min. ^c^ Residual weight percentage at 800 °C in N_2_.

**Table 3:** Experimental and Calculated Oxidation Potentials and Energy Levels of PIs.

	Solution(nm) ^a^		Film(nm)	Oxidation	Potional(V)	E*_g_* ^c^	HOMO ^d^	LUMO ^e^
Polymer	Abs. Max	Abs. onset	Abs. Max	E_onset_	E_1/2_^b^			
TPA-BIA-PI	293	465	318	0.96	1.11	2.67	−4.21	−1.54
TPA-BIB-PI	345	471	344	1.05	1.13	2.63	−4.19	−1.56

^a^ The polymer concentration was 10^−5^ mol/L in NMP. ^b^ E_1/2_ (Average potential of the redox couple peaks). ^c^ The data were calculated by the equation: E*_g_* = 1240/λ_onset_ (energy gap between HOMO and LUMO). ^d^ The HOMO energy levels were calculated from CV and were referenced to ferrocene (4.8 eV; onset = 0.52V). ^e^ LUMO = HOMO − E*_g_*.

## Data Availability

Not applicable.

## Supplementary Materials

The following are available online at https://www.mdpi.com/article/10.3390/molecules28052029/s1, Figure S1: ^1^H–^1^H COSY spectrum of TPA-BIA-NO_2_(a); H–H COSY of TPA-BIB-NO_2_ (b), Figure S2: ^1^H–^1^H COSY spectrum of TPA-BIA-NH_2_(a); H–H COSY of TPA-BIB-NH_2_ (b), Figure S3: FTIR spectra of TPA-BIA-NO_2_ and TPA-BIA-NH_2_, Figure S4: FTIR spectra of TPA-BIB-NO_2_ and TPA-BIB-NH_2_, Figure S5: The oxidation pathways of polyimides TPA-BIB-PI and TPA-BIB-PI, Figure S6: Polyimides with different structures, Table S1: Comparison with other reported polyimide data.

## Sample Availability

Samples of the compounds are available from the authors.
