# Supplementary material for: Synthesis and Characterization of Novel Triphenylamine—Containing Electrochromic Polyimides with Benzimidazole Substituents
Source: Molecules. 2023 Feb 21;28(5):2029. doi: 10.3390/molecules28052029 (PMC10004704; doi:10.3390/molecules28052029)
Supplement: Supplementary file 1 [file molecules-28-02029-s001.zip › molecules-2191172-supplementary.pdf]

Supporting Information

Synthesis and Characteristics of Novel TPA-containing Electrochromic Polyimides with Benzimidazole Substituents

Synthesis of the polyimide

Polymer TPA-BIA-PI: FT-IR(ATR): 1784 cm^{-1} (C=O asymmetric stretching vibration peaks), 1725cm^{-1} (C=O symmetric stretching vibration peaks), 1375cm^{-1} (C-N stretching vibration peaks). ^{1}H NMR (400 MHz, Chloroform-*d*, δ, ppm):8.04 (d, *J* = 8.1 Hz, 1H), 7.95 (s, 1H), 7.86 (dd, *J* = 8.0, 3.5 Hz, 1H), 7.52 (ddd, *J* = 13.4, 7.1, 4.7 Hz, 3H), 7.41-7.28 (m, 3H), 7.32-7.16 (m, 2H), 7.13-7.06 (m, 1H), 1.28 (d, *J* = 23.2 Hz, 1H), 0.84 (s, 1H).

Polymer TPA-BIB-PI: FT-IR(ATR): 1784 cm^{-1} (C=O asymmetric stretching vibration peaks), 1725cm^{-1} (C=O symmetric stretching vibration peaks), 1375cm^{-1} (C-N stretching vibration peaks). ^{1}H NMR (400 MHz, Chloroform-*d*, δ, ppm):8.03 (d, *J* = 8.0 Hz, 2H), 7.95 (s, 2H), 7.85 (d, *J* = 8.1 Hz, 2H), 7.72 (s, 1H), 7.53 (dd, *J* = 19.2, 7.9 Hz, 6H), 7.37-7.14 (m, 20H), 5.35 (s, 1H), 2.23 (q, *J* = 7.7, 6.9 Hz, 1H), 2.01 (d, *J* = 6.6 Hz, 1H), 1.28 (d, *J* = 22.6 Hz, 13H), 1.04 (s, 2H), 0.89 (d, *J* = 6.1 Hz, 1H), 0.85 (s, 6H).

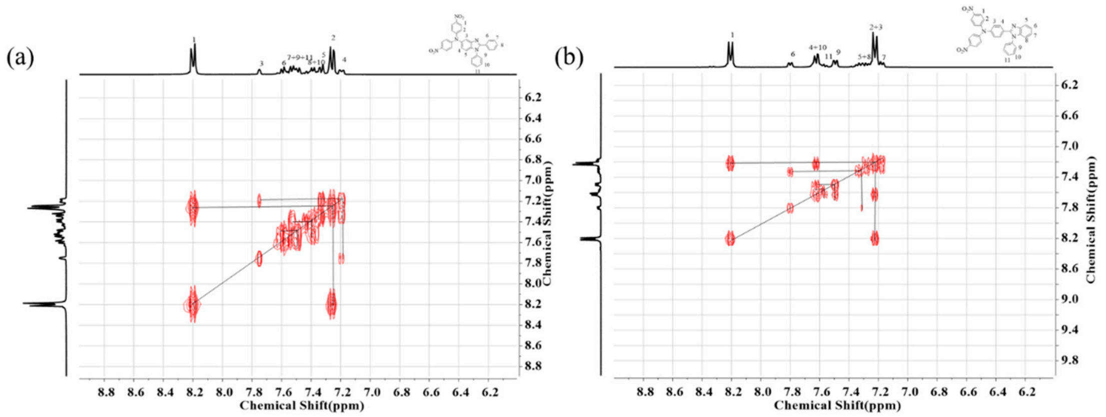

Figure S1. ^{1}H–^{1}H COSY spectrum of TPA-BIA-NO_2(a); H–H COSY of TPA-BIB-NO_2 (b).

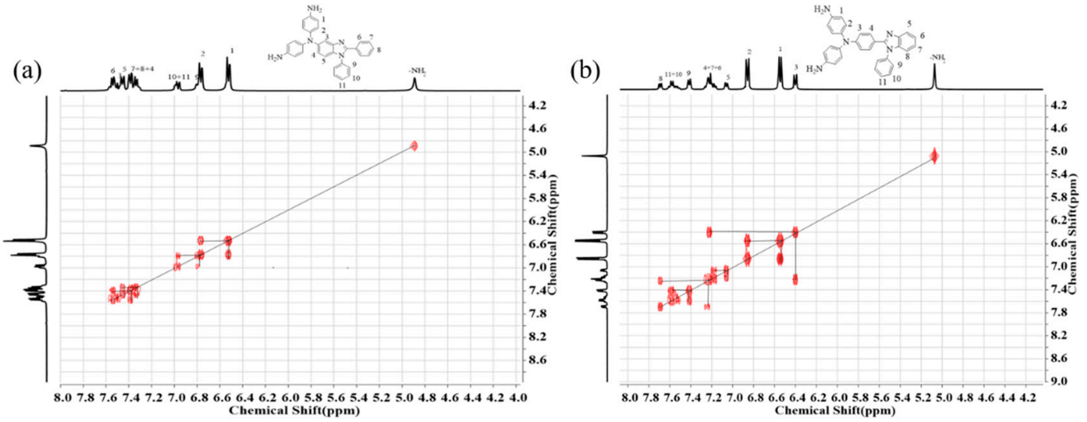

Figure S2. ^{1}H–^{1}H COSY spectrum of TPA-BIA-NH_2(a); H–H COSY of TPA-BIB-NH_2 (b).

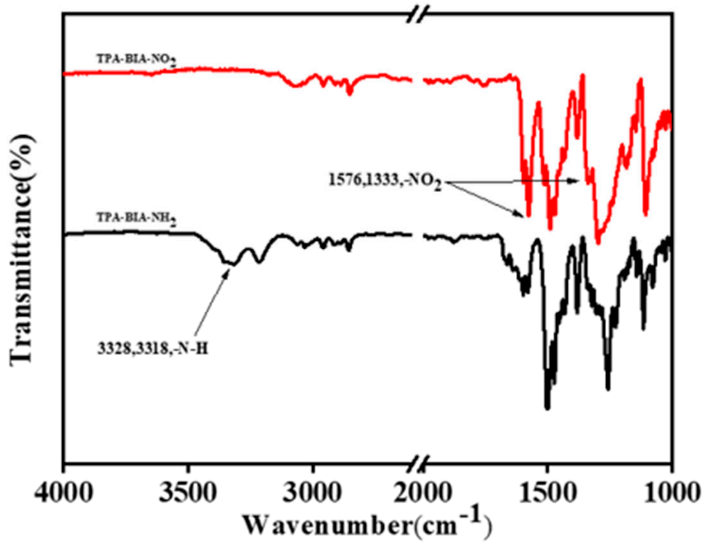

Figure S3. **FTIR spectra of TPA-BIA-NO$_2$ and TPA-BIA-NH$_2$**

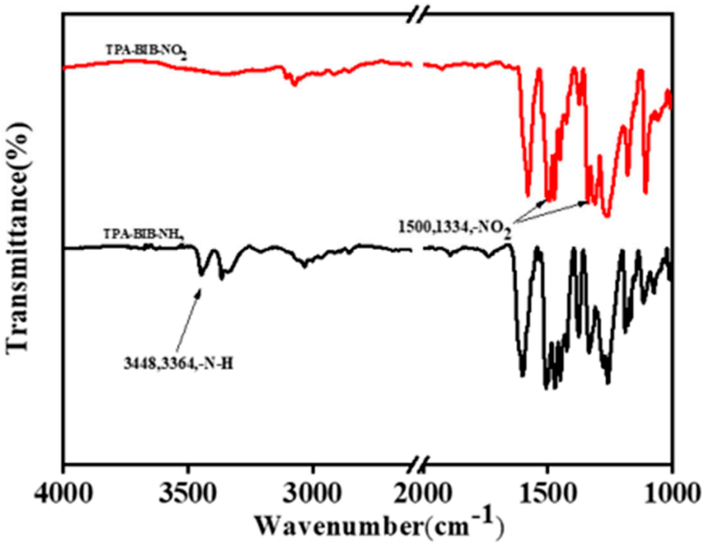

Figure S4. **FTIR spectra of TPA-BIB-NO$_2$ and TPA-BIB-NH$_2$**

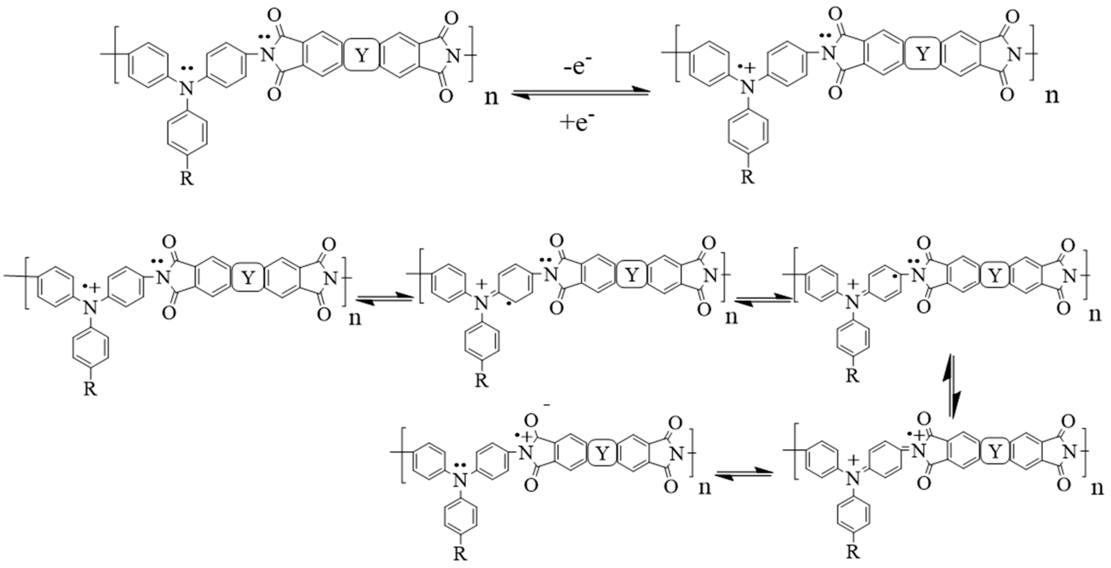

Figure S5. The oxidation pathways of polyimides TPA-BIB-PI and TPA-BIB-PI

Table S1. Comparison with other reported polyimide data[1,2]

	GPC[c]($\times 10^4$ g/mol)			T_g	$T_{d5\%}$	R_{w800}(%)	Oxidation Potional(V)		E_g
**Sample**	**M_w**	**M_n**	**PDI**				**E_{onset}**	**$E_{1/2}$**	
**TPA-BIA-PI**	**15.3**	**12.7**	**1.2**	**343**	**529**	**64**	**0.96**	**1.11**	**2.67**
**TPA-BIB-PI**	**15.0**	**5.8**	**2.6**	**311**	**531**	**67**	**1.05**	**1.13**	**2.63**
**Pe**	**1.1**	**1.7**	**1.6**		**378**	**52**	**0.82**	**0.94**	**3.02**
**MeFPI-3f**	**6.9**	**4.6**	**1.5**	**302**		**65**		**1.04**	**3.10**

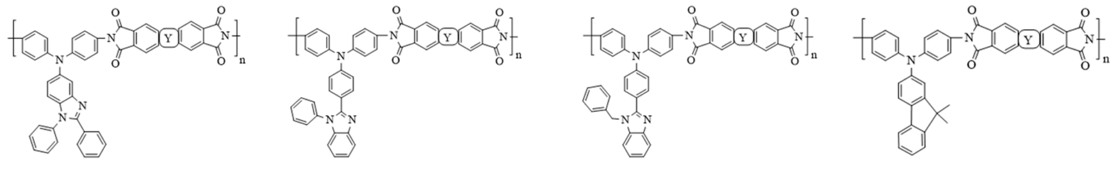

TPA-BIA-PI TPA-BIB-PI Pe MeFPI-3f

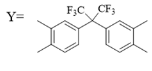

Figure S6. Polyimides with different structures

References

1. Cai, W.; Cai, J.; Niu, H.; Xiao, T.; Bai, X.; Wang, C.; Zhang, Y.; Wang, W. Synthesis and Electrochromic Properties of Polyimides with Pendent Benzimidazole and Triphenylamine Units. *Chin J Polym Sci* **2016**, *34*, 1091–1102, doi:10.1007/s10118-016-1833-1.

2. Sun, N.; Meng, S.; Feng, F.; Zhou, Z.; Han, T.; Wang, D.; Zhao, X.; Chen, C. Electrochromic and Electrofluorochromic Polyimides with Fluorene-Based Triphenylamine. *High Performance Polymers* **2017**, *29*, 1130–1138, doi:10.1177/0954008316671183.
